# Supplementary material for: DNA stable isotope probing reveals the impact of trophic interactions on bioaugmentation of soils with different pollution histories
Source: Microbiome. 2024 Aug 7;12:146. doi: 10.1186/s40168-024-01865-2 (PMC11305082; doi:10.1186/s40168-024-01865-2)
Supplement: Supplementary file 2 — Additional file 1: Figure S1: Normalized DNA concentration according to the density of each fraction for both ST (green lines) and LT (blue lines). Each point represents the mean concentration of the triplicates of c13treatment (dashed lines) and c12control (continuous lines). Red areas indicate density ranges of heavy and light pools. Figure S2: PAH concentrations in ST (A) and LT (B) non inoculated soil microcosms through incubation period. Results are expressed as the mean concentration with their standard deviation. (ACY: acenaphthylene; FLU: fluorene; ANT: anthracene; PHE: phenanthrene; PYR: pyrene; FLN: fluoranthene; B[a]A: benzo(a)anthracene; CRY: chrysene; B[a]P: benzo(a)pyrene). Table S1: Samples identified as outliers by PAH specie, marked by an X, and excluded from the statistical analysis to determine differences in PAH concentration through time. (ACY: acenaphthylene; FLU: fluorene; ANT: anthracene; PHE: phenanthrene; PYR: pyrene; FLN: fluoranthene; B[a]A: benzo(a)anthracene; CRY: chrysene; B[a]P: benzo(a)pyrene). Table S2: Predominant ASV (relative abundance > 0.05%) with the highest percentage of identity with Sphingobium sp. AM 16S rRNA gene sequence. Table S3: Predominant ASV (relative abundance > 0.05%) with the highest percentage of identity with Burkholderia sp. Bk 16S rRNA gene sequence. Figure S3: Correlation between the relative abundance of the ASVs with high identity with the 16S rRNA gene sequence of Sphingobium AM (A) and Burkholderia Bk (B) strain using Spearman correlation method. All the correlations had a p-value < 0.05. Figure S4: Relative abundance of the top 15 bacterial genera in (A) ST and (B) LT non inoculated soil microcosms. Results are expressed as the mean relative abundance of triplicates at time 0 and 15 days or 30 days of incubation for ST and LT soils respectively. Table S4: Kendall correlation coefficient for the enriched bacterial genera abundance regarding Burkholderia and Sphingobium abundances in both ST and LT soils [file 40168_2024_1865_MOESM1_ESM.docx]

**Supplemental material**


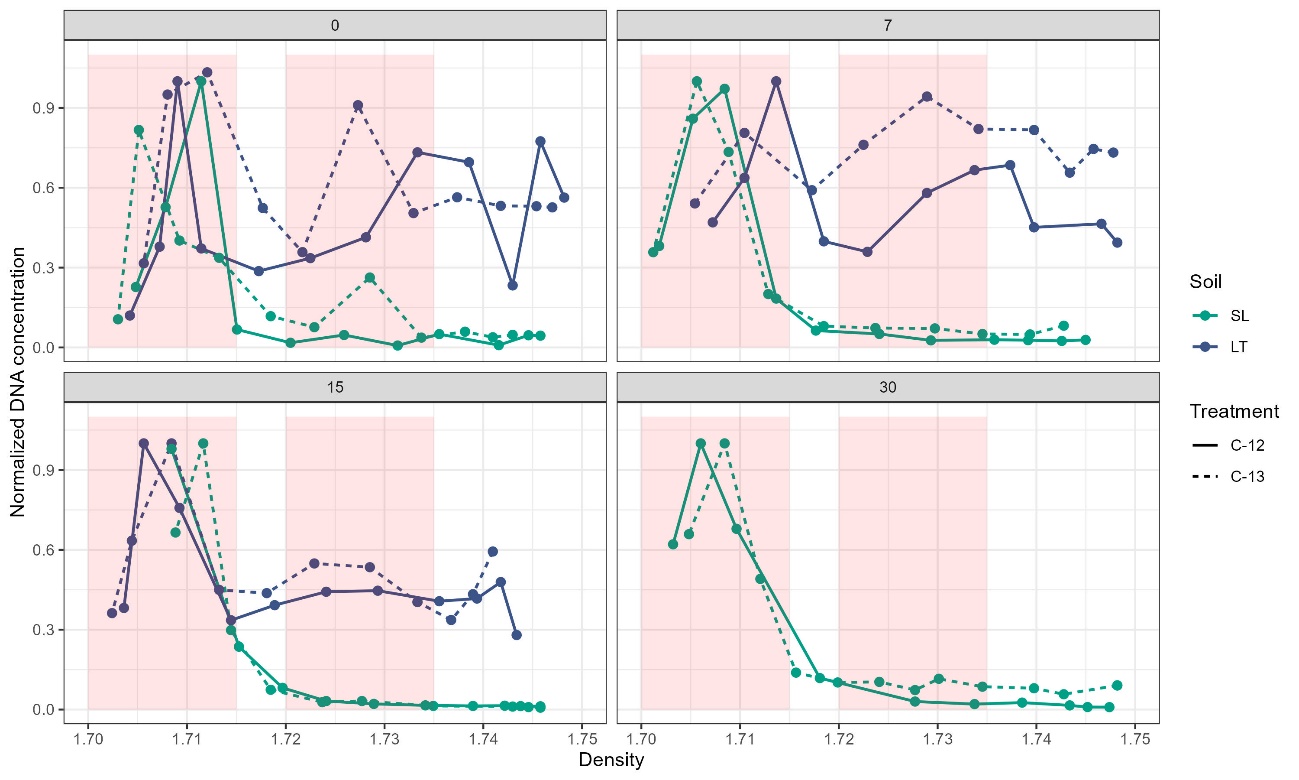


Figure S1: Normalized DNA concentration according to the density of each fraction for both ST (*green lines*) and LT (*blue lines*). Each point represents the mean concentration of the triplicates of ^c13^treatment (*dashed lines*) and ^c12^control (*continuous lines).* Red areas indicate density ranges of heavy and light pools.


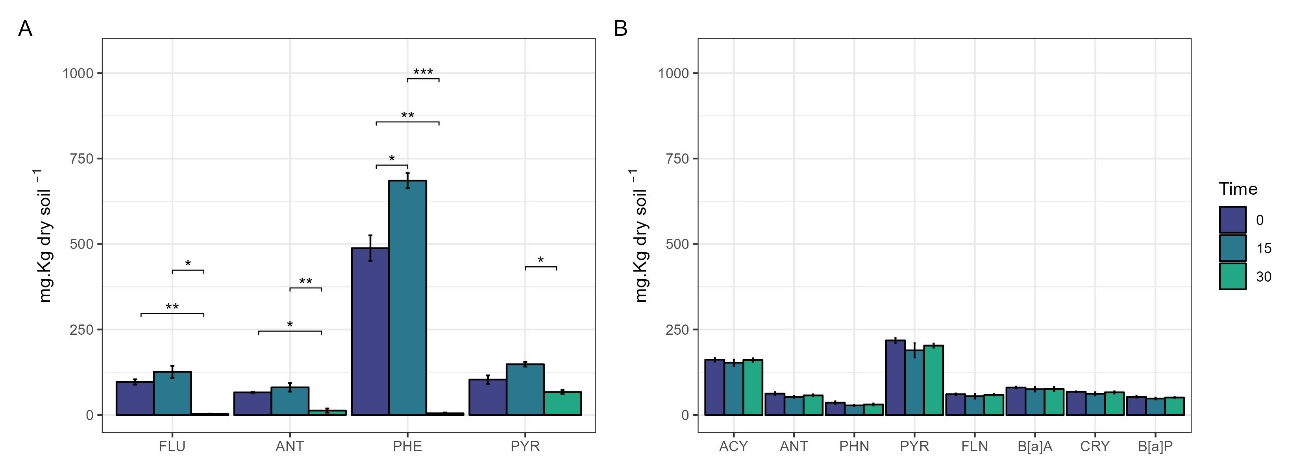


Figure S2: PAH concentrations in ST (A) and LT (B) non inoculated soil microcosms through incubation period. Results are expressed as the mean concentration with their standard deviation. (ACY: acenaphthylene; FLU: fluorene; ANT: anthracene; PHE: phenanthrene; PYR: pyrene; FLN: fluoranthene; B[a]A: benzo(a)anthracene; CRY: chrysene; B[a]P: benzo(a)pyrene).

| Sample | ID |  | | | | | | | | |
| --- | --- | --- | --- | --- | --- | --- | --- | --- | --- | --- |
|  |  | ACY | FLU | ANT | PHN | PYR | FLN | B[a]A | CRY | B[a]P |
| ^12^C Control ST | 1 |  |  |  |  | **X** |  |  |  |  |
| ^12^C Control ST | 2 |  |  |  |  |  |  |  |  |  |
| ^12^C Control ST | 3 |  |  |  |  |  |  |  |  |  |
| ^13^C Treatment ST | 4 |  |  |  |  |  |  |  |  |  |
| ^13^C Treatment ST | 5 |  |  |  |  |  |  |  |  |  |
| ^13^C Treatment ST | 6 |  |  |  |  |  |  |  |  |  |
| ^12^C Control LT | 1 |  |  |  |  |  |  | **X** |  | **X** |
| ^12^C Control LT | 2 | **X** |  |  |  |  | **X** |  |  |  |
| ^12^C Control LT | 3 |  |  |  |  |  |  |  |  |  |
| ^13^C Treatment LT | 4 |  |  |  | **X** |  |  |  | **X** |  |
| ^13^C Treatment LT | 5 |  |  |  |  |  |  |  |  |  |
| ^13^C Treatment LT | 6 |  |  | **X** |  | **X** |  |  |  |  |

Table S1: Samples identified as outliers by PAH specie, marked by an **X,** and excluded from the statistical analysis to determine differences in PAH concentration through time. (ACY: acenaphthylene; FLU: fluorene; ANT: anthracene; PHE: phenanthrene; PYR: pyrene; FLN: fluoranthene; B[a]A: benzo(a)anthracene; CRY: chrysene; B[a]P: benzo(a)pyrene).

| ASV | % identity |
| --- | --- |
| ASV4  ASV3 | 99.762  99.524 |

Table S2: Predominant ASV (relative abundance > 0.05%) with the highest percentage of identity with *Sphingobium* sp. AM 16S rRNA gene sequence.

| ASV | % identity |
| --- | --- |
| ASV2  ASV76  ASV70  ASV1  ASV69  ASV58 | 100.000  99.775  99.775  99.775  99.551  99.551 |

Table S3: Predominant ASV (relative abundance > 0.05%) with the highest percentage of identity with *Burkholderia* sp. Bk 16S rRNA gene sequence


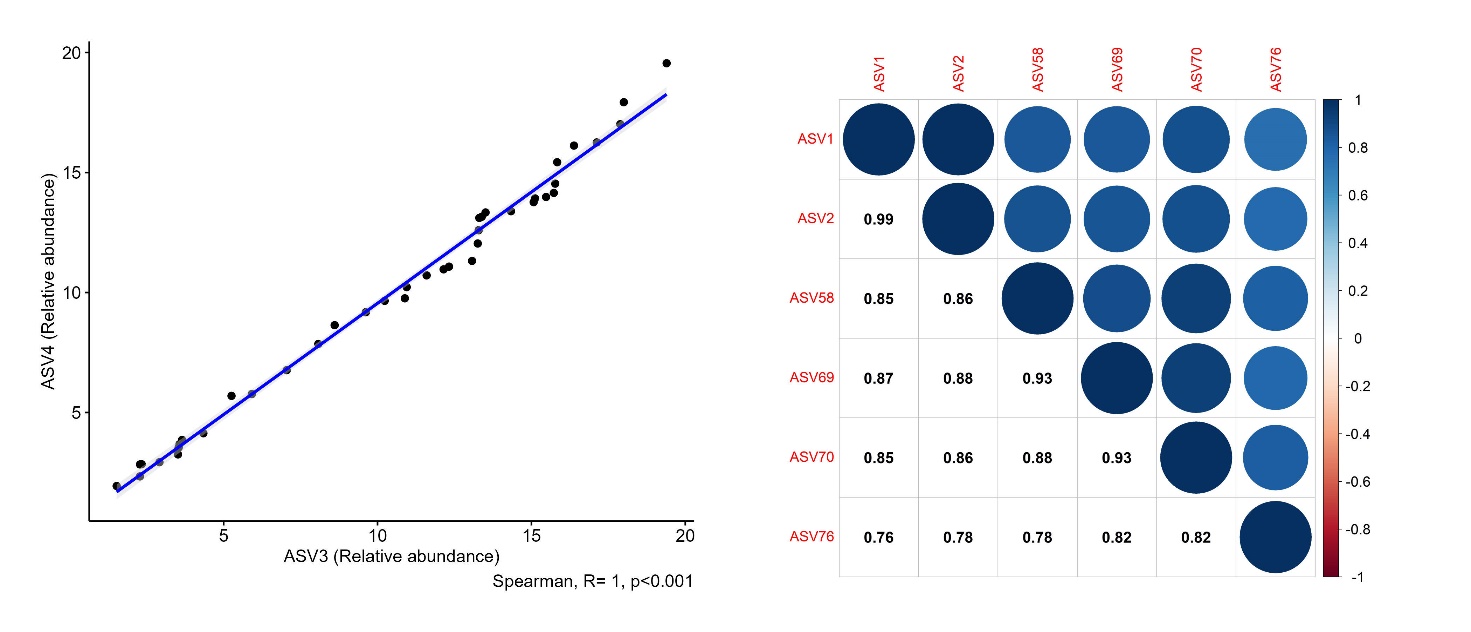


B

A

Figure S3: Correlation between the relative abundance of the ASVs with high identity with the 16S rRNA gene sequence of *Sphingobium* AM (A) and *Burkholderia* Bk (B) strain using Spearman correlation method. All the correlations had a p-value < 0.05.


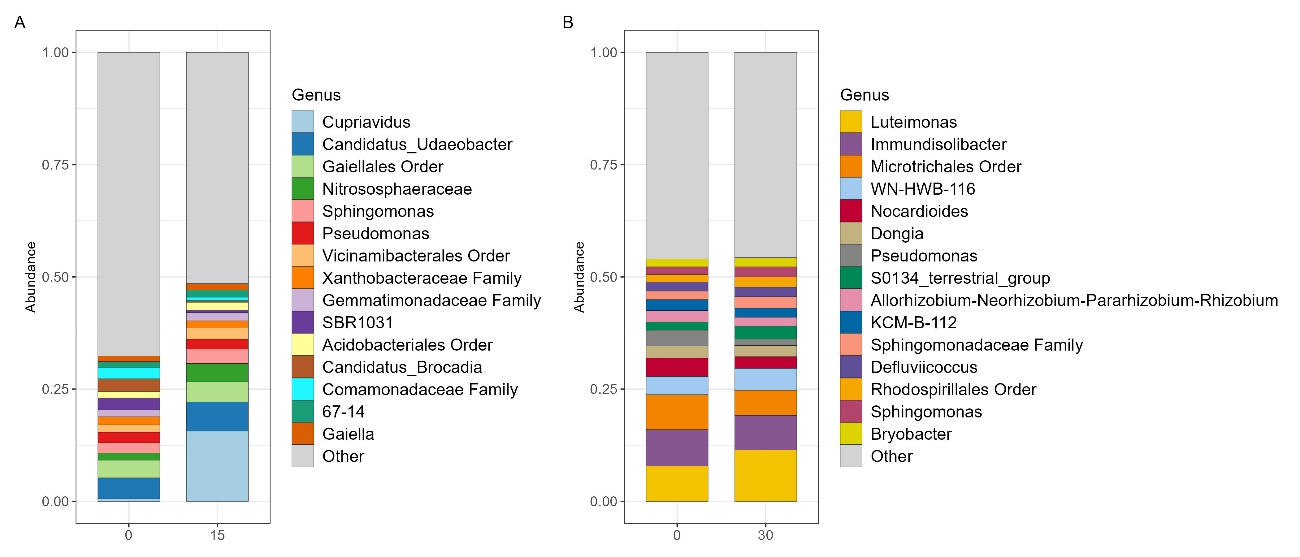


Figure S4: Relative abundance of the top 15 bacterial genera in (A) ST and (B) LT non inoculated soil microcosms. Results are expressed as the mean relative abundance of triplicates at time 0 and 15 days or 30 days of incubation for ST and LT soils respectively.

|  | Correlation with Burkolderia abundance | Correlation with Sphingobium abundace |
| --- | --- | --- |
| Sphingomonas | -0,76*** | -0,82*** |
| Bradyrhizobium | -0,64*** | -0,82*** |
| Acidobacteriales | -0,82*** | -0,82*** |
| Rokubacteriales | -0,58** | -0,82*** |
| Subbroup_2 | -0,79*** | -0,79*** |
| Gemmatimonas | -0,73** | -0,79*** |
| Candidatus_Udaeobacter | -0,88*** | -0,76*** |
| Lysobacter | -0,79*** | -0,73** |
| Mycobacterium | -0,57** | -0,73* |
| Xanthobacteraceae | -0,48* | -0,73*** |
| Vicinamibacteraceae | -0,67** | -0,73*** |
| Candidatus_Solibacter | -0,60** | -0,72** |
| Methyloligellaceae | -0,67** | -0,70** |
| RB41 | -0,79** | -0,67** |
| Gemmatimonadaceae | -0,52* | -0,64** |
| Bacillus | -0,64** | -0,64** |
| Subgroup_7 | -0,69** | -0,63** |
| Rhodanobacter | -0,53* | -0,59* |
| Acidothermus | -0,42 | -0,55* |
| TK10 | -0,51* | -0,54* |
| WN-HWB-116 | -0.42 | -0.50 |
| 67-14 | -0.57 | -0.50 |
| Promicromonospora | -0.50 | -0.48 |
| Chitinophagaceae | -0,36 | -0,48* |

Table S4: Kendall correlation coefficient for the enriched bacterial genera abundance regarding Burkholderia and Sphingobium abundances in both ST and LT soils. **p <* 0.05, ***p <* 0.01, ****p <* 0.001.

|  | Correlation with Burkolderia abundance | | Correlation with Sphingobium abundace |
| --- | --- | --- | --- |
| Allas | | -0,71** | -0,75** |
| Cercomonas | | -0,67** | -0,38 |
| Acanthamoeba | | -0,56 | -0,35 |
| Sorodiplophrys | | -0,31 | -0,09 |
| uncultured Techofilosea | | -0,24 | -0,16 |
| Telaepolella | | -0,16 | -0,16 |
| D3P05A02 | | -0,09 | -0,24 |
| Sorosphaerula | | 0,05 | 0,13 |
| Filamoeba | | 0,05 | -0,16 |
| uncultured_eukaryote | | 0,05 | 0,13 |
| Mortierella | | 0,24 | 0,31 |
| uncultured Agaricales | | 0,35 | 0,35 |
| uncultured Cercomonadidae | | 0,38 | 0,31 |
| LKM11 | | 0,56* | 0,64** |
| Hygrocybe | | 0,67** | 0,67** |

Table S: Kendall correlation coefficient for the enriched eukaryotic genera abundance regarding Burkholderia and Sphingobium abundances in ST soil. **p <* 0.05, ***p <* 0.01, ****p <* 0.001.

|  | Correlation with Burkolderia abundance | | Correlation with Sphingobium abundace |
| --- | --- | --- | --- |
| D3P05A02 | | -0,43 | -0,64* |
| Spumella | | -0,36 | -0,29 |
| Lobochlamys | | -0,29 | -0,21 |
| Colpoda | | -0,07 | -0,29 |
| Opisthonecta | | 0,24 | 0,24 |
| Chlamydomyxa | | 0,43 | 0,21 |
| Filamoeba | | 0,79 | 0,57 |
| D3P05A02 | | -0,43 | -0,64 |

Table S5: Kendall correlation coefficient for the enriched eukaryotic genera abundance regarding Burkholderia and Sphingobium abundances in LT soil. **p <* 0.05, ***p <* 0.01, ****p <* 0.001.
